# Emerging Penetrating Neural Electrodes: In Pursuit of Large Scale and Longevity

**DOI:** 10.1146/annurev-bioeng-090622-050507

**Published:** 2023-06-08

**Authors:** Lan Luan, Rongkang Yin, Hanlin Zhu, Chong Xie

**Affiliations:** 1 Department of Electrical and Computer Engineering, Rice University, Houston, Texas, USA; 2 Rice Neuroengineering Initiative, Rice University, Houston, Texas, USA; 3 Department of Bioengineering, Rice University, Houston, Texas, USA

**Keywords:** electrophysiology, penetrating electrodes, large scale, chronic

## Abstract

Penetrating neural electrodes provide a powerful approach to decipher brain circuitry by allowing for time-resolved electrical detections of individual action potentials. This unique capability has contributed tremendously to basic and translational neuroscience, enabling both fundamental understandings of brain functions and applications of human prosthetic devices that restore crucial sensations and movements. However, conventional approaches are limited by the scarce number of available sensing channels and compromised efficacy over long-term implantations. Recording longevity and scalability have become the most sought-after improvements in emerging technologies. In this review, we discuss the technological advances in the past 5–10 years that have enabled larger-scale, more detailed, and longer-lasting recordings of neural circuits at work than ever before. We present snapshots of the latest advances in penetration electrode technology, showcase their applications in animal models and humans, and outline the underlying design principles and considerations to fuel future technological development.

## INTRODUCTION

Nerve cells, or neurons, generate electrical signals to transmit information across the brain and through the body. This intriguing property has motivated scientists and engineers to develop technologies to electrically interrogate and actuate the nervous system. Taking the brain as an example of the nervous system, electrical interfaces can be divided into three categories on the basis of the placement of the electrical contacts and the implantation strategy: (*a*) electrodes noninvasively placed on the scalp to record an electrogram that represents the macroscopic activity of the brain underneath, for electroencephalograms (1); (*b*) grid or strip electrodes placed on the brain surface directly under the dura mater or within a sulcus, for electrocorticography (ECoG) (2); and (*c*) penetrating electrodes that are invasively implanted into the brain tissue (3, 4). Among these various electrical interfaces, penetrating neural electrodes have two unique capabilities: First, they can detect the voltage signals from a small volume of the tissue in a specific brain region in the vicinity of the electrodes, often deep into the brain rather than restrained on the brain surface. Second, they can tune in to individual neurons at locations on demand, providing time-resolved detections of individual action potentials and their orchestrated activity in neuronal ensembles. These capabilities have led to powerful approaches to decipher and augment the nervous system, enabling fundamental understandings of functions of specific neurons and subregions, as well as the development of human prosthetic devices that restore crucial functions such as sensation and movement. This review focuses on microscale penetrating electrode technology that improves the ability to detect and track individual neurons in the brain. It also includes a small subset of non-penetrating approaches that share common design principles with their penetrating counterparts. Noninvasive devices are beyond the scope of this review. Integrated circuits and mechanisms to transmit signals from and to the penetration electrodes are included when they are part of the electrode design. Specialized algorithms to process these signals are not the focus of this review and have been discussed in other reviews (5, 6).

Action potentials from individual neurons produce large transmembrane potentials in the vicinity of their somata. Recording extracellular action potentials (also referred to as single-unit spikes) requires microscopic recording sites, of sizes similar to or smaller than a cell soma, to be implanted into a targeted brain region to achieve the spatial proximity of the neurons of interest. The recording sites thus transduce signals that arise from the current flow through the extracellular space during the propagation of action potentials, providing high temporal resolution samplings of individual neurons in the close vicinity of the contacts. Because neurons of the same class in the same region generate identical action potentials, the only way to putatively identify a given neuron is by the spike amplitude waveform morphology, which changes as a function of distance and direction from the neuron (7, 8). Importantly, the ability to detect and resolve individual neurons, often referred to as unit isolation quality, deteriorates significantly as the distance from the electrode increases and diminishes at approximately 50 μm (8). Therefore, approaches to improve the efficacy of extracellular single-unit recording have centered on two strategies: (*a*) reducing the distance between the recording sites and neurons to tune into a specific neuron(s), and (*b*) employing more electrodes to record from more neurons and to dissociate spikes from different nearby neurons. For example, a microdrive moves the tip of a tungsten microwire substantially closer to the cell body of one neuron to achieve high-quality unit isolation (9). Building upon the success of tungsten microwires (3), stereotrodes (10) and tetrodes (11) provide two or four recording sites in close proximity, allowing for the triangulation of distances between the spike-emitting cell bodies and the recording sites. Taking advantages of microelectromechanical systems (MEMS) technology in the last few decades, silicon microelectrode arrays (MEAs) (Michigan electrodes) (12) have overcome the inherent channel-number limitations of microwires and substantially increased the number of recording sites per implantation site. These penetrating microelectrodes have enabled temporally resolved monitoring of local neural circuits and have made prominent contributions to neuroscience, as summarized in previous review articles (4, 8, 13).

Neural recordings using penetration microelectrodes are invasive, and this characteristic imposes two major challenges. The first challenge is scalability to a large number of recording sites: The desire for large-scale recordings that are able to monitor many neurons in a small volume competes with the desire to minimize the tissue damage imposed by the electrodes (14). The second challenge is longevity: While it is straightforward to achieve the spatial proximity between neurons and electrodes by inserting microelectrodes into the brain tissue, maintaining the spatial proximity and stability over time is challenging. Implantation of devices into the brain is most often accompanied by foreign body responses at the tissue–electrode interface, which leads to instability and deterioration of recordings.

In this review, we discuss technological advances in the past 5–10 years that have overcome these challenges to enable larger-scale, more detailed, and longer-lasting recordings of neural circuits at work than ever before. We loosely divide the content into two sections—electrode development focusing on large-scale recordings and on longevity, respectively—but we note that the challenges and solutions are often interwoven. In the first section, we discuss two approaches: passive and active devices to significantly increase the channel count and channel density of penetration electrodes. In the second section, we outline the multifaceted considerations and achievements of improving recording stability and lasting durations. In both sections, we include applications in animal models and humans to showcase the unique capabilities of recordings enabled by these technological advancements. Altogether, this review presents snapshots of the latest advances in penetration electrode technology and outlines the underlying design principles to fuel future technological development.

## EMERGING TECHNOLOGY FOR LARGE-SCALE RECORDINGS

### General Considerations of Large-Scale Recordings

The distributed coding theory, in which coordinated activation of neurons at multiple locations within the same brain region and across a large number of different brain areas underpins sensation (15, 16), memory (17), decision (18, 19), and action (20, 21), has accumulated an increasing amount of physiological evidence in recent years. The theory is further reinforced by the concurrent anatomical discovery of long-range, brain-wide neuronal projections (22–30) that hypothetically mediate the interregional modulation. The capability to densely record neurons from a given area and/or simultaneously record them from a wide range of brain regions is a prerequisite for studying and advancing our understanding of the collaboration mechanisms of spatially dispersed neurons underlying behavior.

Intracortical electrodes can only faithfully detect and distinguish neurons within ~50 μm (8, 31). Therefore, monitoring the activity of a large number of neurons requires a large number of recording sites implanted in the tissue, inevitably risking tissue displacement and injury. Importantly, most of the device volume, which dictates the tissue displacement during implantation, is not occupied by the microscopic recording sites but instead is occupied by the mechanical support of the device and the interconnections that address the recording sites. For instance, Utah or microwire arrays, which have enabled relatively large-scale recordings (hundreds of neurons) from primates (32–35), carry only one recording site on the tip of each wire in a volume of 20–100 μm in diameter and a few millimeters in length. On the other hand, microfabricated silicon electrodes carry arrays of recording sites that are distributed along each shank, but the number of channels is limited by the fabrication resolution of the interconnections for a given width of the device (12).

**Figure 1:**
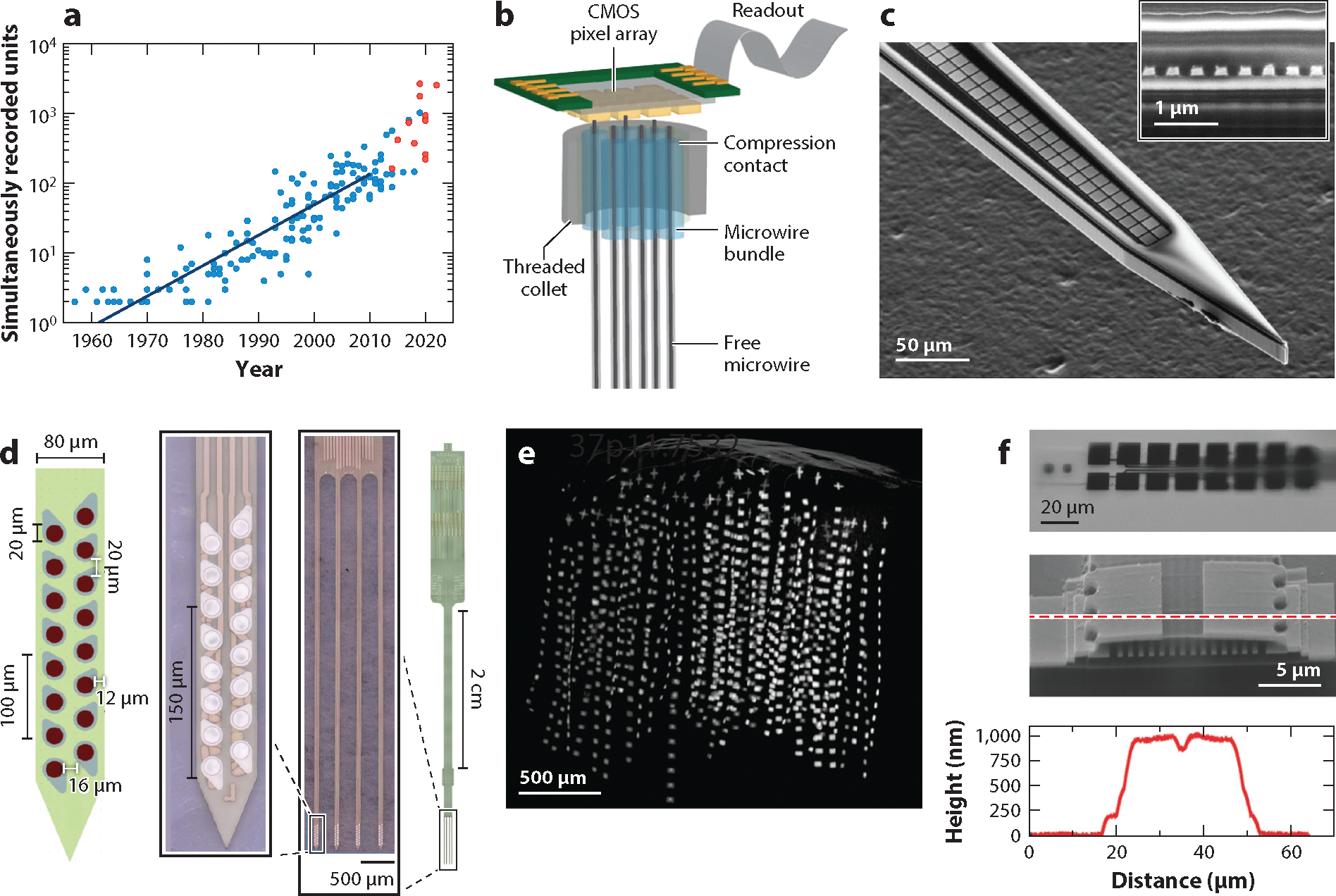
Representative large-scale passive electrodes in the past decade. (*a*) The number of simultaneously recorded neurons has increased with time and accelerated in the past decade. Blue dots represent data adapted with permission from Reference 37 (CC BY 4.0). Red dots represent recent works discussed in this review. (*b*) High-density microwire bundles integrated with a complementary metal-oxide semiconductor (CMOS) pixel array. Panel adapted with permission from Reference 42 (CC BY 4.0). (*c*) Nanofabricated silicon microelectrode array. Image of the cross-sectional area (*inset*) shows interconnects 200 nm in width. Panel adapted with permission from Reference 48. (*d*) A polyimide electrode (16 recording sites per shank, four shanks per device). Co-implantation of 16 devices achieved 1,024-channel recording of freely moving rats. Panel adapted with permission from Reference 51. (*e*) A micro–computed tomography scan showing the volumetric distribution of an 8 × 8 × 16 (*x* × *y* × *z*) (1,024-channel) ultraflexible electrode array in a mouse visual cortex. Panel adapted with permission from Reference 53. (*f*) Photographic and scanning electron microscopy images of a 16-channel ultraflexible electrode fabricated with electron-beam lithography. The cross-sectional graph shows a thickness of 1,000 nm. Panel adapted with permission from Reference 56 (CC BY 4.0).

Generally speaking, there are three orthogonal axes to increase the recording sites without aggravating the tissue damage during implantation: (*a*) reducing the volume of the device, particularly the volume of the substrate or mechanical support; (*b*) increasing the fabrication resolution to accommodate more interconnections per area; and (*c*) implementing multiplexing circuits to reduce the interconnection needed to address a large number of recording sites. Emerging neural–electrode technologies for large-scale recordings push along individual or multiple directions of the above axes. These efforts have led to a tremendous increase in the number of simultaneously recorded neurons, following an exponential growth curve faster than that of the past decades (36, 37) (Figure 1a). Here, we coarsely divide the technologies into two categories: (*a*) passive electrodes that use the conventional wire-recording site design and (*b*) active electrodes with multiplexing circuits.

### Passive Electrodes at Large Scales

Passive electrodes use the conventional concept of one wire for one sensor. It is possible to scale up the deployment of traditional electrodes (tetrodes and Utah arrays) by implanting multiple instances of the same array. Such electrodes are widely available, and their performance has been well characterized. For example,128 tetrodes (512 available channels) along with two optical fibers were simultaneously implanted in 13 regions in the mouse brain to study brain-wide modulation of firing rate in response to natural fear stimuli and localized optogenetic stimulation (38).Sixteen Utah arrays, 64 channels each, were implanted into the visual cortices of monkeys to map multiunit receptive fields and correspondingly elicit specific shape perception by stimulating neurons around selected hundreds of electrodes (39). However, these approaches increased the total volume occupied without increasing the recording density. Furthermore, due to the limited number of channels per implantation module, such an approach often entails lengthy and highly skilled surgical procedures and specially designed connectors/pedestals to route the dense wiring from each individual array to commercial amplifier and recording systems. The routing wires are often too heavy for the animals (at least for rodents) to safely carry without external support.

Deploying highly integrated electronics to transmit the electrical signal and reducing the electrode dimensions circumvent the aforementioned limitations. Carbon-fiber electrodes (CFEs) offer a minimized implantation footprint of <10 μm in diameter per wire and allow for closely spaced arrays for simultaneous recording of unit activity and electrochemical sensing of dopaminergic activity (40). However, scaling is laborious since each wire (or a small number of wires) is fabricated and assembled one at a time. This scaling issue was resolved by bundling individual wires together and directly bonding the wire bundle to a complementary metal-oxide semiconductor (CMOS) amplifier array chip (Figure 1b) adapted from infrared camera sensors (41). The microwire arrays were customized for deep and shallow brain regions, leading to scalable implantations (i.e., 163 microwires) into the rat motor cortex (42). Assisted by a specially designed application-specific integrated circuit, a similar approach further scaled to 65,536 available recording channels sampled at 32 kHz using a 12-bit analog-to-digital converter (43). In vivo demonstration showcased evoked local field potentials (LFPs) from more than 30,000 channels in a sheep implant, and 700 units out of 1,300 channels in a rat implant. As an alternative strategy, dual-side lithographic microfabrication processes were exploited to construct a 1,024-channel penetrating silicon microneedle array (32 × 32 grid) with a flexible backing made of 10-μm-thick polyimide. The device is optically transparent, permitting simultaneous optical and electrophysiological interrogation of neuronal activity (44).

Silicon high-density electrode arrays were among the first to scale up channel amount and spatial density by improving fabrication resolution. Initial efforts to increase available channels fabricated 32 channels per shank and increased the shank number per device to eight, which achieved 256 channels per device (45). Further refinement of MEMS fabrication provided high-yield devices with a minimal feature size of 0.4 μm, resulting in 64 channels in a single shank of 7 mm × 86 μm × 23 μm (*l* × *w* × *t*). In vivo recording in mice with a 16-shank implant showed up to 308 simultaneously recorded units in one animal (46). In a similar study, stacking 16 shanks in a 4 × 4 (*x* × *y*) array allowed construction of a high-density 3D silicon electrode array of 1,024 recording sites in a volume of 0.6 mm^3^ (47). Nanofabrication using electron-beam lithography reduced the minimal feature size, the width of the interconnect, to 200 nm (Figure 1c) and produced 200 closely spaced recording channels per shank (48). More recently, considerable effort has been made to reduce the dimension and rigidity of silicon neural probes to mitigate the severe tissue damage they could cause. Cellular-sized silicon probes with eight recording sites per shank, minimized shank dimensions of 5 μm × 10 μm, and 32 closely spaced shanks at the intershank spacing as small as 66 μm were integrated to provide a total of 256 electrodes per implantation (49).

Flexible electrode arrays, developed to prolong recording durations (discussed in the next section), have also been demonstrated to be on a promising path toward large-scale recordings on par with their rigid counterparts. Integrating 16 polyimide neural probes with a hyperdrive that is commonly used for tetrodes permitted fine positioning of each probe and provided 256 recording sites in freely moving rats (50). A platform integrating flexible polymer electrodes (Figure 1d) using a modular stacking head-stage design supported up to 1,024 recording channels in freely behaving rats and demonstrated months-long recordings from hundreds of well-isolated units across multiple brain regions (51). Reducing the thickness of the flexible probes allows for further increase of the volumetric density. Constructed with ultrathin electrodes that were only 1 μm in thickness (52), an 8 × 8 × 16 (*x* × *y* × *z*) 3D implant in a rat provided 1,024 channels (Figure 1e) and isolated 987 units in a tissue volume of 1 mm^3^, the highest 3D recording density demonstrated by far (53). A separate mouse implant demonstrated 144 recording shanks, 1,930 channels distributed in multiple cortical regions, and resolution of 2,548 units in total. To facilitate implantation of large-scale flexible arrays, multiple high-throughput implantation methods were developed. A surgical robot demonstrated rapid implantation of 32 probes one at a time (54), while parallel insertion of multiple flexible shanks assisted by biodissolvable adhesives allowed implantation of several shanks simultaneously using off-the-shelf, cost-efficient supplies (55). Importantly, the recording site density on the flexible electrodes can be further improved by electron-beam lithography (Figure 1f), making it possible to increase the recording channel counts and density per shank by another order of magnitude without major changes of implantation strategies (56).

In addition to large-scale recording of penetrating electrodes, there are endeavors to perform high-density large-scale micro-ECoG (μECoG) recordings. A 20-μm-thick flexible array of 1,152 electrodes (300 for simultaneous recording) provided a large spatial coverage of 14 × 7 mm^2^ for nonhuman primate recordings (57). Large-scale platinum nanorod grids have densely spaced recording sites at a 150-μm intersite pitch, providing 1,024 channels for rodents and 2,048 channels for human applications. Spatiotemporal propagation of epileptic discharges during human surgery were observed at unprecedented density and scale (58). While spinal cord electrophysiological recordings are relatively uncharted, recently, 60-channel flexible polyimide probes (59) at 4.5-μm thickness were implanted into cat dorsal root ganglions for acute recordings, demonstrating great potential for large-scale high-density recordings in the spinal cord and simultaneous large-scale recordings in both the brain and the spinal cord.

### Active Electrodes with Multiplexing Circuits

**Figure 2:**
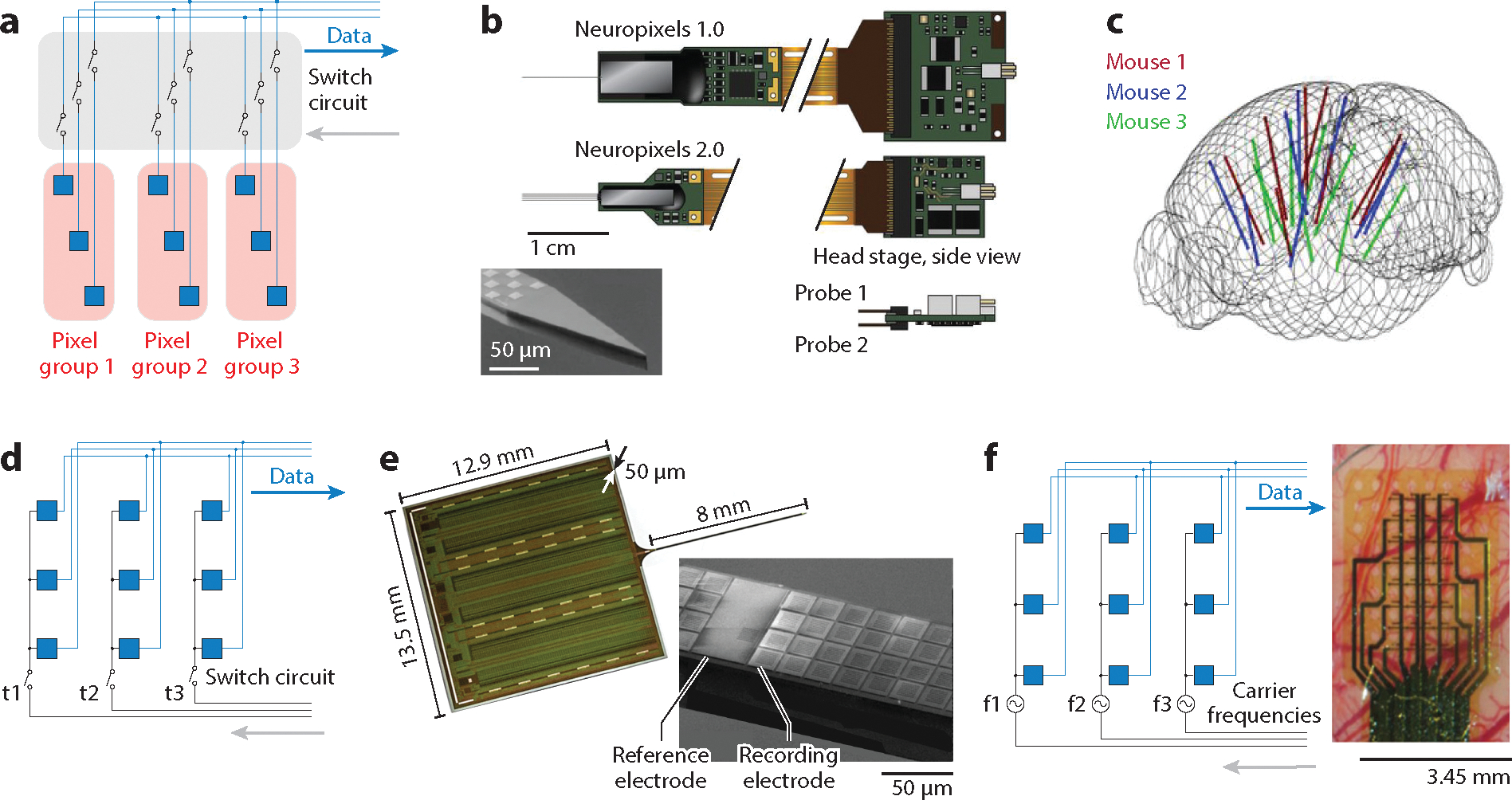
Active electrodes with three types of multiplexing circuits: channel selection, time division, and frequency division. (*a*) A simplified circuit diagram for channel-selection multiplexing. Recording channels are organized into pixel groups. One pixel group is selected prior to experiments for recording by the switch circuit. (*b*) Neuropixels probes as representative examples of channel-selection multiplexing. The dimension and the design of the recording shank for the Neuropixels 1.0 are shown in the inset. The Neuropixels 2.0 further reduces the head-stage size while enabling the integration of two probes (four shanks per probe) on a single head stage. Panel adapted with permission from References 62 and 63. (*c*) Reconstructed implantation location from three mice, each implanted with eight Neuropixels 1.0 probes. This design gives the highest simultaneous recorded unit yield (2,668) reported to date. Panel adapted with permission from Reference 21. (*d*) A simplified circuit diagram for time-division multiplexing. Each row of the recording sites shares a single data readout line. Only one column is activated by the switch circuit at a given time point, and each column is activated sequentially and periodically. (*e*) A representative time-division multiplexing probe of 1,356 recording channels. Panel adapted with permission from Reference 72 (CC BY 4.0). (*f*) (*left*) A simplified circuit diagram for switchless frequency-division multiplexing. Each row of recording sites shares a single data readout line. They are modulated by different carrier frequencies and are demodulated to allow simultaneous readout of low-frequency to DC signals by later-stage signal processing circuits. (*right*) A representative frequency-division multiplexing probe with 32 recording channels on a rat brain for ultraslow wave recording. Panel adapted with permission from Reference 77a.

A major advantage arising from incorporating active circuits within neural recordings is their capability of multiplexed recording. Multiplexing can further be divided into time-division multiplexing, frequency-division multiplexing, and channel-selection multiplexing. For a more detailed circuit classification and description, see the review by Guimerà-Brunet et al. (60). Using channel-selection multiplexing (Figure 2a) and advanced 130-nm CMOS microfabrication technology (61), Neuropixels incorporated 960 densely integrated channels on a single shank with a cross section of 70 × 20 μm (Figure 2b). The active switching circuit under each recording site enabled selection of 384 simultaneous recording channels, offering diverse spatial coverage along the implantation depth (62). Recently, the Neuropixels 2.0 (63) increased the number of implantable shanks per probe from one to four and the number of available recording channels per shank from 960 to 1,280 (64). Only one head stage was required to record from two such quad-shank probes, which resulted in 10,240 recording channels in one compact implant, out of which 768 channels can simultaneously record (63). The standardized and scalable CMOS fabrication technology enabled mass production at a reasonable cost and facilitated broad dissemination. Simultaneous implantation of multiples of such high-density probes has driven extremely large-scale recordings in animals. Six Neuropixels probes implanted in six different visual areas permitted the study of across-region functional connection hierarchy (65), whereas eight Neuropixels probes (Figure 2c) with more than 3,000 channel recordings deciphered the brain-wide synchronous response to thirst signals (21). The experiment recorded 1,462 to 2,668 units at approximately 0.48 to 0.87 units per channel. Albeit some degree of loss in recorded neurons occurred, Neuropixels probes supported chronic recording of a few months (66) and allowed for reimplantation after explantation (67). Notably, Neuropixels probes have fueled large-scale single-unit recording in human subjects (68, 69).

In addition to increasing recording channel counts, channel-selection multiplexing enables versatile recording for electrodes that switch between the detection of different types of molecules (70) or electrodes that switch between recording AC high-frequency LFPs and DC infraslow signals (71).

In the scheme of time-division multiplexing, recording sites are arranged into M groups, which are sampled simultaneously through M digitization circuits, and N sites per group, which are scanned sequentially (Figure 2d). The number of wires needed to address M × N sites are reduced to *O*(*M* + *N*). Because penetration electrodes typically sample at 20–30 kHz to resolve action potentials, 50- to 100-fold times higher than the sampling frequency of μECoG arrays, they require 50- to 100-fold more wires to address the same number of recording sites. Using time-division multiplexing, the NeuroSeeker array (Figure 2e) recorded from 1,356 channels on one shank at 20 kHz, but with an elevated noise level (72). In the same vein, multi-shank simultaneous neural active pixel sensor (SiNAPS) probes (73) offered 1,024 recording channels in a total of four shanks and demonstrated large-scale recording of light-evoked spiking and LFP response (74). The device architecture of SiNAPS probes differs from that of Neuropixels and NeuroSeeker probes in that the amplifier circuit is beneath each pixel (active pixel sensor), which reduces the total silicon surface area used by the probe and base combined (75). Flexible polyimide electrodes using a 16:1 multiplexing ratio provided 64 densely patterned recording sites per shank at a shank size of 60 × 4 μm (*w* × *t*), with four shanks and 256 channels in total for high-density localized recordings (76).

Frequency-division multiplexing (77) could also reduce the number of connection lines while eliminating the noise induced by switching circuits in time-division multiplexing. In this scheme (Figure 2f), the DC to low-frequency signal in the N electrodes is converted to N spectrally separated carrier frequencies by amplitude modulation to be simultaneously recorded in one single channel. M demodulation circuits at the back end then give a readout and are converted back to M × N signals. Using a 4 × 8 (*x* × *y*) graphene solution–gated field-effect transistor (g-SGFET) electrode array in frequency-division multiplexing mode, visually evoked LFPs and ultraslow-frequency cortical spreading depolarization in rats were recorded (77a). The unique capacity for g-SGFET arrays to record DC signals (71, 77a, 78) opens up new clinical translation opportunities where multiple pathological biomarkers manifest in ultraslow electrical signals (79) that have been traditionally ignored by the overwhelming number of AC recording technologies.

## EMERGING TECHNOLOGY TO IMPROVE RECORDING LONGEVITY

### General Considerations of Long-Lasting Single-Unit Recordings

Extending high-fidelity electrophysiological studies of brain circuits to span diverse time scales from days to years is critical for understanding the neural dynamics and plasticity underlying learning, aging, and the progression of neurological disorders. However, it is still a great challenge to achieve reliable chronic recordings of single units. For example, large-scale human studies using the only intracortical microelectrode array cleared by the US Food and Drug Administration for humans—the Utah array—showed that more than 50% of implants had a unit yield [defined as having a signal-to-noise ratio (SNR) > 1.5] per electrode of less than 40% after a year of implantation (80). Moreover, both acute and chronic single-unit recordings showed significant variations that confounded the decoding of neural activity (81, 82).

In attempts to improve the recording quality, the failure modes of the penetration electrodes, including both biotic and abiotic factors, have been extensively studied (83–85). Biotic factors, such as immune responses from acute and chronic foreign body reactions (FBRs) in the brain tissue and meninges, are major contributors to the diminishing quality of unit recordings over time. The implantation of electrodes initiates sustained FBRs. The progression of FBRs produces reactive oxygen species, glial encapsulation of the implanted electrodes, and neuronal degeneration, leading to subsequent loss of recording efficacy (86). Furthermore, these adverse tissue responses induce a corrosive environment (87) and accelerate the structural and material degradation of electrodes, resulting in, for example, delamination and corrosion of the insulation layers (88). As a result of the interactions between these biotic and abiotic factors, recording quality, particularly the ability to resolve individual neurons, typically deteriorates over several weeks (85, 89–92) and is often accompanied by mechanical and structural failures of the electrodes (93–96).

Given the crucial role of FBRs from both the biotic and abiotic perspectives, alleviating device-induced FBRs is a key consideration in achieving reliable chronic recordings. There are generally two promising strategies for developing immune-evasive neural interfaces. The first strategy is based on the hypothesis that the mechanical mismatch between the MEA and the tissue is the main cause of sustained FBRs; thus, this strategy seeks to design tissue-compliant devices to minimize FBRs. The second strategy focuses on intervening in the FBR process directly using antibiofouling coatings and local anti-inflammation drug delivery. In the following section, we review the emerging technologies that exploit these two strategies to improve chronic stability of the neural interface.

### Mechanical Mimesis

Neural probes are often tethered on the skull of the subject while the recording sections are inserted into the brain tissue. The brain tissue is separated from the skull by the dura mater. The gap is filled with cerebrospinal fluid and is approximately 100 μm in mice and 2–4 mm in humans (97). The floating brain tissue is constantly moving due to respiration, pulsations, and rapid head movements. In the rat brain, the micromotions from respiration, of a magnitude of ~20 μm, account for most of the stress, while the contribution from pulsatile vasculature is minuscule (2–4 μm) (98). Due to the mechanical mismatch in the interface between the probe and the tissue, the relative movement exerts an enhanced stress on the tissue, resulting in sustained FBRs. In the meantime, the built-up strain in the probes accelerates cracking and delamination of the insulating material.

The efforts to bridge the mechanical mismatch in the interface between the probe and the tissue are largely divided into two veins: (*a*) reducing the dimension and bending stiffness of the device by controlling the form factors and (*b*) fabricating devices from very soft materials with an elastic modulus (or Young’s modulus) similar to the viscoelastic brain tissue. Both approaches have shown decreased neuronal death and glial encapsulation and extended stable recordings.

#### Reducing dimensions and bending stiffness.

When a neural probe is simplified as a cantilevered beam, the bending stiffness *K* is the ratio between the longitudinal loading force and the displacement. The bending stiffness *K* comprehensively characterizes the mechanical interactions at the probe–tissue interface when the probe is deflected due to relative motions within the tissue (52, 99). *K* scales linearly with the Young’s modulus of the material and, importantly, has a strong dependence on the physical dimensions of the device, which we discussed in detail in a previous review (100). Briefly, *K* is proportional to the smallest dimension of the device to a high power; for example, *K* is proportional to (thickness)^3^ in a slab geometry, as in silicon MEAs, and to (diameter)^4^ for a cylindrical beam, as in microwires (101). Therefore, reducing the size along the smallest dimension of the device provides an effective way to significantly reduce *K* and tamp down FBRs.

**Figure 3:**
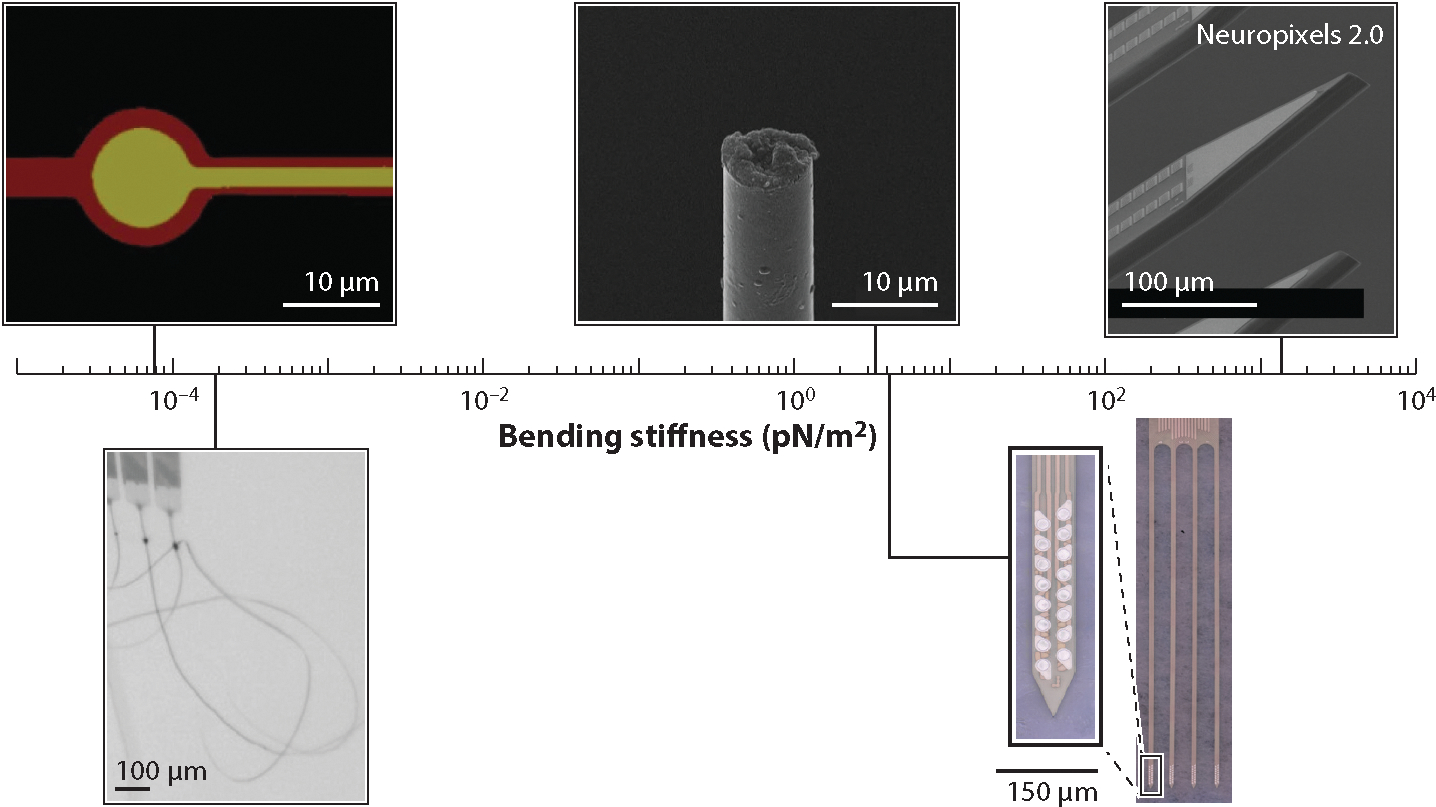
Emerging neural electrodes and their bending stiffnesses. From left to right: NeuE (neuron-like electronics) (104), NET-10 (nanoelectronic thread) (52), carbon-fiber electrodes (106, 108), polyimide probe (51), and Neuropixels 2.0 (63, 64). Images of representative electrodes reproduced with permission.

One of the most effective ways to reduce the thickness of an electrode and lower bending stiffness is to remove the mechanical support completely. This was demonstrated in both a mesh (99, 102–104) and a shank (52, 56) geometry at a total thickness of 1 μm using photoresist SU8 as the insulation layer without an additional substrate (Figure 3). Postmortem histology (99, 102, 104) and in vivo longitudinal imaging (52, 56) of neurons, astrocytes, microvasculature, and microcirculation revealed normal neuronal density, an intact blood–brain barrier (BBB), and few to no glial responses near these ultraflexible electrodes in rodents. Consistently, recording efficacy, including single-unit yield and SNR, was several months long (52, 102) without noticeable degradation. In an effort to further reduce the bending stiffness, a free-standing gold nanofilm electrode was developed (105). The nanofilm electrode forms an intimate and conformal interface with neural tissues and records single units for 3 months without a polymer substrate support (105).

Parallelly, ultrasmall carbon-fiber electrodes (CFEs), with diameters of 5.5–8.4 μm including the insulation layer, reduce the dimension by approximately one order of magnitude compared with that of traditional microwires and demonstrate much reduced FBRs with minimal to nonexistent glial scarring (106–108). The ultrasmall size even makes the CFEs record intracellular-like action potentials in songbird auditory forebrain nuclei (107). Moreover, a 16-channel array of CFEs recorded single-unit activity for at least 3 months, with average amplitudes of ~200 μV (108). Additionally, carbon nanotube fibers provide an alternative carbon-based ultrasmall neural electrode, which is softer than CFEs and has a lower impedance, making it a potential candidate for chronic recording (109, 110).

It is worth noting that all these studies of ultrasmall and ultraflexible neural probes have, so far, been performed in mice and rats. While they consistently show excellent integration with brain tissue and long-lasting recordings, long-term mechanical and electrical durability and tissue responses in larger animals have yet to be assessed.

#### Softness.

**Figure 4:**
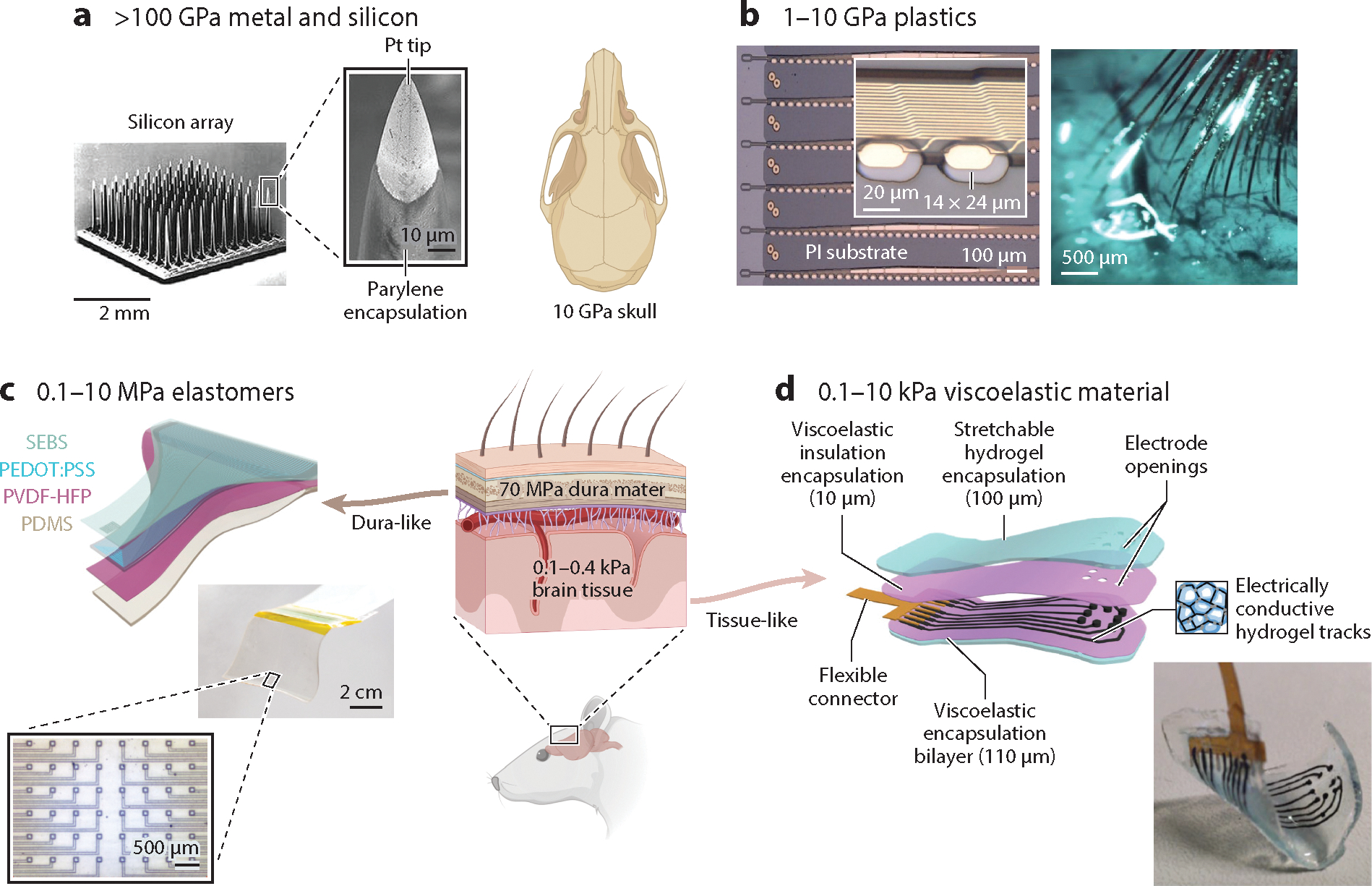
A comparison of the Young’s moduli of conventional and emerging neural electrodes. (*a*) Scanning electron microscopy images of a Utah array. The zoomed-in view shows the platinum (Pt)-coated silicon tip and the parylene-coated shaft. Panel adapted with permission from Reference 112. (*b*) Images of the polyimide (PI)-based thread electrodes and their dense implantation in a rat cortex. The inset shows a zoomed-in view of the electrodes. These electrodes are made of plastic or silicon and have Young’s moduli comparable to or larger than that of the skull. Panel adapted with permission from Reference 54. (*c*) Topological supramolecular network–enabled stretchable electrodes, including an exploded view (*top left*), an image of the assembled device (*middle*), and a zoomed-in view of the contact (*bottom left*). Panel adapted with permission from Reference 113. (*d*) Viscoelastic electrode array, including an exploded view (*top*) and an image of the fully assembled array (*bottom right*). Panel adapted with permission from Reference 114.

As an orthogonal alternative to minimizing the size or the thickness of the probes, choosing softer constituent materials is another approach to reduce FBRs. The Young’s modulus *E* is the comprehensive mechanical metric of softness. For silicon, *E* is approximately 150–170 GPa, at least six orders of magnitude higher than that of brain tissue (3–100 kPa; neurons and astrocytes reported at 1–100 Pa) (111) (Figure 4a). Finite-element analysis comparing a rigid silicon probe (165 GPa), a flexible polyimide probe (10 GPa), and a softer probe (200 kPa) with the same geometry revealed a 94% reduction in the strain value at the tip of the polyimide probe in the tissue relative to the silicon probe (91). Moreover, the softer probe (200 kPa) induced two orders of magnitude smaller values of strain compared with the silicon probe (91), indicating that softer substrates reduce the strain at the probe–tissue interface and thus may reduce tissue response in chronic implants.

Plastic polymers such as polyimide, SU8, and parylene C have become popular materials to construct flexible devices (see previous review in 100). However, the Young’s moduli of these plastics are still in the range of gigapascals and several orders of magnitude higher than those of brain tissues (Figure 4b). Furthermore, brain tissues exhibit both viscous and elastic behavior under stress and strain and need to be considered as viscoelastic materials. Therefore, a class of ultrasoft viscoelastic materials has been explored to construct neural implants.

Elastomers are viscoelastic, are intrinsically stretchable, and have a Young’s modulus lower than dura mater. A neural electrode based on elastomers was developed by molecular engineering of a topological supramolecular network to achieve high mechanical robustness and high electrical conductivity (113) (Figure 4c). The device could control organ-specific activities through the rat brain stem at single-nucleus precision. Hydrogel is another viscoelastic material with a Young’s modulus in the kilopascal range, approaching the softness of brain tissue. A surface microelectrode array, composed of a hydrogel-based conductor made from an ionically conductive alginate matrix enhanced with carbon nanomaterials and an insulation layer made of self-healing polydimethylsiloxane (PDMS), demonstrated recording of auditory evoked potentials from a rat cortex (114) (Figure 4d). Another study integrated fibers in a soft hydrogel matrix to construct a multifunctional soft device with adaptive bending stiffness that showed minimal FBRs and the ability to track stable isolated single units in freely moving mice over 6 months (115).

While viscoelastic bioelectronics can accommodate repeated strain induced by the dynamic movement of the organ, they could assert substantial stress to the organ during this process. By fine-tuning the polymer properties, a morphing electrode (MorphE) (116), consisting of viscoplastic polymers that were permanently deformed by slow tissue growth, was developed. MorphE enabled conformal adaptation to sciatic nerve growth while causing minimal damage to the rat nerve during the fastest growth period, allowed chronic electrical stimulation and monitoring for 2 months without disruption of functional behavior, and offered a promising material strategy for interfacing with developing nervous systems and other organs.

Shape memory polymers (SMP) are another category of polymers for adjusting the softness of the neural probe. There has been recent progress in achieving fabrication of intracortical MEAs from a SMP substrate that remains stiff at room temperature but softens after implantation to 20 MPa, which is an order of magnitude softer than previously reported SMP-based devices (117). Such devices demonstrated chronic recordings and electrochemical measurements for 16 weeks in rat cortices. Meanwhile, they are robust to physical deformation during implantation, facilitating surgical implementation.

A key challenge for soft materials is the risk of compromised electrical and mechanical stability in vivo. For example, swelling of hydrogel-based polymers in the hydrated state results in pushing target neurons away from the electrode; increased porosity leads to failed insulation and conductivity chronically (118). To overcome this challenge, the choice of cross-linker type and cross-linking density need to be fine-tuned to adjust for the appropriate mechanical and electrical stability before long-term intracortical applications.

### Antibiofouling Coatings and Local Anti-Inflammation Drug Delivery

**Figure 5:**
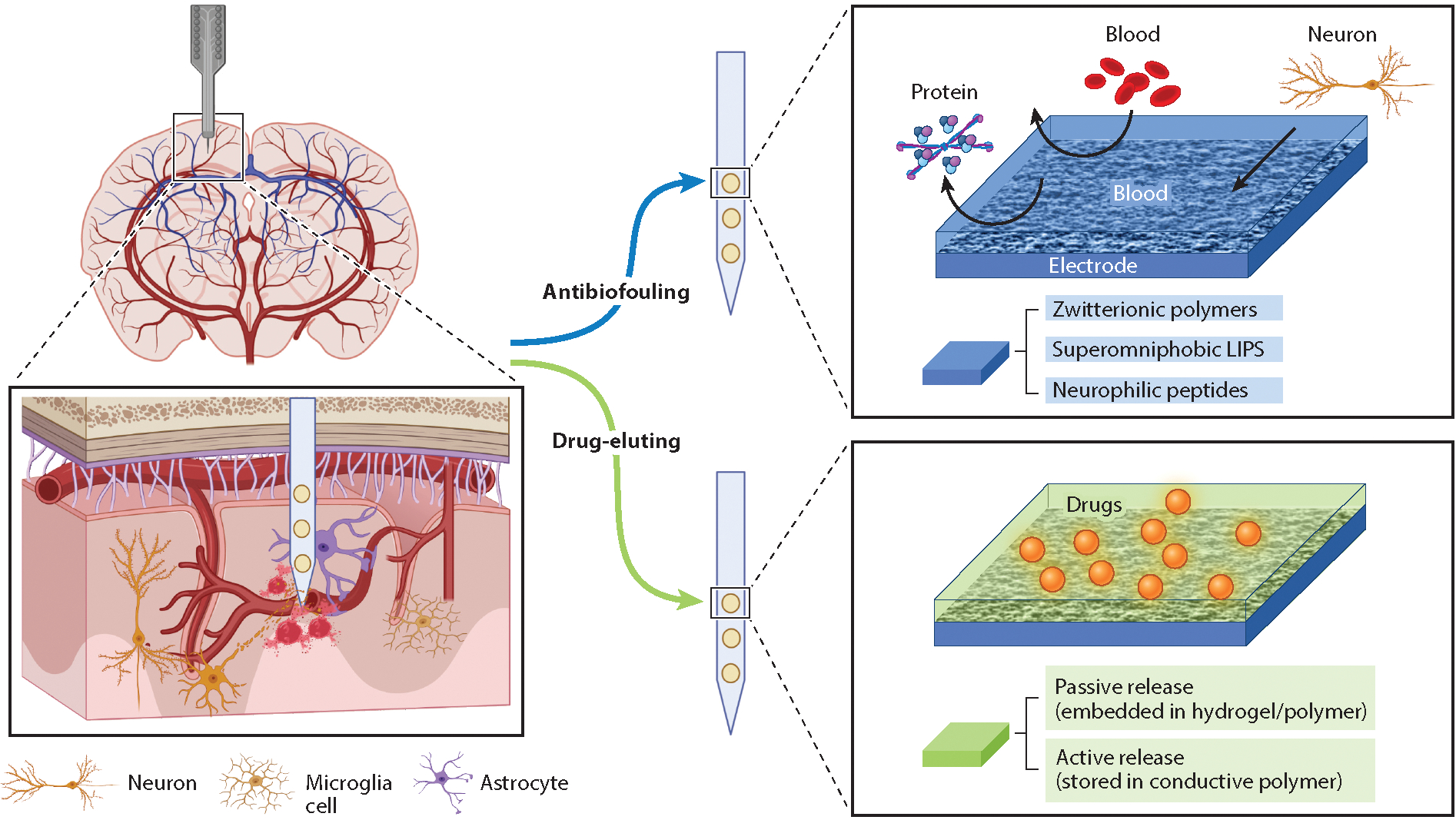
Schematics of the strategies to suppress foreign body reactions by manipulating the local chemical environment: antibiofouling coatings and local anti-inflammation drug delivery. Antibiofouling coating strategies include zwitterionic polymers, superomniphobic liquid-infused porous surfaces (LIPS), and neurophilic peptides. Anti-inflammation drug delivery strategies include passive and active release.

The insertion of electrodes into the brain tissue inevitably causes tissue damage, including blood vessel ruptures and breakage of the BBB, which are regarded as two of the culprits for initiating FBRs (119). Although theoretically it is possible to avoid damaging vascular networks altogether during implantation, it is yet to be demonstrated, given that the estimated mean distance of a neuronal soma to the closest capillaries is only ~15 μm in a mouse brain (120). BBB damage will lead to leakage of proteins such as albumin and fibrinogen, which nonspecifically adsorb to the surface of the electrodes. These proteins serve as a tag for immune cells to recognize the electrodes as a foreign body and initiate the acute inflammation process, followed by a cascade of cellular event that form FBRs (119). Therefore, a variety of approaches have been developed to minimize the absorption of the extravasated blood proteins on the electrodes or to suppress the downstream inflammatory responses (Figure 5).

#### Antibiofouling coatings.

The most used antibiofouling polymers include poly(ethylene glycol) (PEG) and PEGylated polymers (97). As a hydrophilic material, PEG forms a tightly bound hydration layer with water molecules via hydrogen bonds. The hydration layer acts as a physical barrier to prevent absorption of biomolecules. However, coating neural electrodes with PEG showed no significant suppression of FBRs at 4 weeks in mice, despite the reported acute antibiofouling property (121).

Zwitterionic polymers have recently attracted great attention. Zwitterionic polymers have an equal amount of anionic and cationic groups in their monomer unit. The coexistence of the oppositely charged groups confers a high dipole moment and high polarity to the polymer, leading to high hydrophilicity and the formation of a hydration layer through ion solvation on the probe interface, thus making it capable of blocking out biomolecules. In vivo two-photon imaging in CX3CR1-GFP mice of a neural probe coated with a synthetic zwitterionic polymer [poly(sulfobetaine methacrylate)] shows that the zwitterionic coating significantly suppresses the microglial encapsulation of neural electrodes in the acute FBR phase (122). Additionally, zwitterionic peptides were engineered to have two functions (123): to create a hydration layer that resists protein adsorption on the microelectrodes, and to increase the adhesion of the microelectrodes to neuronal cells. This zwitterionic peptide modification of flexible electrodes showed stable recording for 16 weeks and greatly reduced neuron loss in mice (123).

There are other alternatives in addition to creating a hydration layer. With a focus solely on promoting neuron adhesion, L1, a neuronal-specific cell adhesion molecule, was covalently bound to a silicon probe surface, resulting in high single-unit yield for 16 weeks in the V1m cortex of mice (124). Along the opposite direction, inspired by Nepenthes pitcher plants, an antifouling approach was developed by creating a slippery liquid-infused porous surface to achieve stable superomniphobicity, which repels both polar and nonpolar molecules and creates a near-frictionless surface (125). Fabricating such an antifouling surface on a silicon-based neural probe successfully reduced the implantation trauma and prolonged the duration of single-unit recordings to 16 weeks, compared with a period of 8 weeks using noncoating probes (126).

#### Anti-inflammatory drug delivery.

The design of anti-inflammatory drug-eluting neural probes is another potential promising direction for inhibiting FBRs and increasing neural recording longevity. As one of the most prominent corticosteroid drugs that inhibits proinflammatory signals and has an immune-suppressant effect, dexamethasone is commonly released from polymer coatings to suppress FBRs. To enable the fine control of dosing over chronic periods, an actively controlled method was developed. The drug was stored in a conducting polymer coating (PEDOT/dexamethasone) on the neural probes and was released weekly by the application of a cyclic voltammetry signal. The inhibition of FBRs was evaluated for 12 weeks in rats, and an overall attenuated immune response was reported (127). However, the delivery of dexamethasone not only suppresses immune response but also often affects neuronal repair (128). Another inflammasome inhibitor, MCC950, demonstrated potential to overcome this drawback. MCC950 was incorporated into the silicone coating of the electrodes for local delivery in vivo, which reduced FBRs in peripheral nerve injury implant models without affecting neuronal repair. This therapeutic approach to prevent FBRs holds promise for adapting to the central nervous system and prolonging the functional life span of neural probes (129).

## FUTURE PERSPECTIVES

Neural activity in the brain and other nervous systems spans an enormous dynamic range spatiotemporally, from fast dynamics at submilliseconds to experience-dependent plasticity over years and decades, and from subcellular structural and functional changes to whole-brain synchrony. Therefore, understanding the nervous system requires the development and use of synthetic tools to cross these diverse temporal and spatial scales. Penetrating neural electrodes have traditionally been the most powerful in detecting the fast dynamics of small numbers of sparsely distributed neurons. The various strategies discussed in this review have enabled numerous new opportunities for understanding neural circuits at work by increasing the number of neurons simultaneously monitored and enabling longer-lasting periods of high-efficacy monitoring.

Novel electrode technology must disseminate to broad user groups to maximize its impact. Integrating the perspectives of electrode development and application, we propose a figure of merit (FOM) of electrode values as the cost per spike it records throughout its lifetime. Under the coarse approximation that all channels have the same spike recording efficacy, $FOM=n/c\int_{0}^{T}\;y(t)dt$, where n is the total channel in the electrode, c is the resource (material, labor) cost of manufacturing, T is the total implantation lifetime, and y(t) is the average spike yield per channel as a function of implantation time. The emerging approaches to increase recording scale and improve longevity must achieve these goals in a cost-effective and scalable manner to make the strongest impact. For example, the academic use of Neuropixels is surging, thanks to the standardized CMOS fabrication technology that has enabled mass production of these high-recording-scale devices at a reasonable cost. Several of the flexible electrodes, including 1-μm nanoelectronic threads (52, 53) and polyimide electrodes (51), were fabricated using standard photolithography or ebeam lithography and therefore have similarly low fabrication cost.

The interface between penetrating electrodes and electronics that filter, amplify, digitalize, and transmit the recorded neural signal to computers is an integral part of the device, and thus all aspects of this interface must be optimized. The tethered electrode-electronics interface must be compact, scalable, and robust for hundreds to thousands of connecting-disconnecting cycles, or more, to support the ever-growing need for recording at large scales and over chronic implantation periods. Most conventional connectors fall short on either density or robustness. Electrodes with integrated, miniaturized electronics can circumvent this issue.

It is worth noting that most of the approaches have demonstrated success only in specific applications, regions, and species. The broad application of these emerging technologies will require nontrivial optimization to adapt to other brain regions and species by accounting for the significant anatomical and functional differences. This process takes time and demands high resources. Quantitative metrics that rigorously evaluate the electrode functions across multiple testing labs are necessary to guide the optimization. Furthermore, most of the strategies focus on one particular design consideration and aim for one-knob control of the complex, multifaceted process that dictates recording efficacy in vivo. A holistic design, which integrates coherent strategies to optimize multiple orthogonal parameters such as materials, form factor, assembly, packaging, and implantation methods, is likely to provide the most effective path toward long-term stable recording of large numbers of neurons.
